# Factors Associated with Chronic Chikungunya in Vitória, Espírito Santo State, Brazil, Between 2016 and 2020

**DOI:** 10.3390/v16111679

**Published:** 2024-10-28

**Authors:** Creuza Rachel Vicente, Luana Santos Louro, Nicolli Ribeiro de Jesus, Danielle Torres dos Santos Lopes, Aline Souza Areias Cabidelle, Crispim Cerutti Junior, Angelica Espinosa Barbosa Miranda, Iuri Drumond Louro, Debora Dummer Meira, Kuan Rong Chan

**Affiliations:** 1 Department of Social Medicine, Federal University of Espírito Santo, Vitória 29047-105, Espírito Santo State, Brazil; fil.cris@terra.com.br (C.C.J.); angelica.miranda@ufes.br (A.E.B.M.); 2 Post-Graduate Program in Infectious Diseases, Federal University of Espírito Santo, Vitória 29047-105, Espírito Santo State, Brazil; nicollirj@gmail.com (N.R.d.J.); danitsl@yahoo.com.br (D.T.d.S.L.); 3 Medical School, Federal University of Espírito Santo, Vitória 29047-105, Espírito Santo State, Brazil; luana.louro@edu.ufes.br; 4 Health Surveillance Service, Department of Health, Vitória 29017-010, Espírito Santo State, Brazil; areiasaline927@gmail.com; 5 Department of Animal Biology, Federal University of Espírito Santo, Vitória 29075-910, Espírito Santo State, Brazil; iuri.louro@ufes.br (I.D.L.); debora.dummer.meira@gmail.com (D.D.M.); 6 Post-Graduate Program in Biotechnology, Federal University of Espírito Santo, Vitória 29047-105, Espírito Santo State, Brazil; 7 Program in Emerging Infectious Diseases, Duke-NUS Medical School, Singapore 169857, Singapore

**Keywords:** chikungunya fever, chronic pain, arthralgia, clinical epidemiology

## Abstract

Chikungunya patients may develop chronic joint pain that can persist for months to years. This study aimed to determine the factors associated with Chikungunya chronicity. This case–control study involved data from patients with laboratory-confirmed Chikungunya reported from March 2016 to December 2020 in Vitória, Espírito Santo state, Brazil. The data were accessed from compulsory notification databases (SINAN and eSUS VS) and electronic medical reports (Rede Bem-Estar). For each patient who developed chronic symptoms, we included a control patient who did not develop chronic symptoms by random sampling. A total of 183 chronic and 183 non-chronic patients were included in the study. Most of them were female (73.2%), with a median age of 49.5 years (interquartile range = 37–61), and presented fever (89.6%), myalgia (89.6%), arthralgia (89.3%), and headache (82.0%). Chronic patients were older (median = 53; interquartile range = 41–61) than non-chronic cases (median = 46; interquartile age = 31–61) (OR = 0.979, 95% CI = 0.968–0.991) and more frequently presented nausea (58.5% vs. 40.4%; OR = 2.109, 95% CI = 1.374–3.238), and leukopenia (20.2% vs. 10.9%; OR = 2.060, 95% CI = 1.122–3.779). Therefore, these characteristics should be monitored for the better clinical management of cases prone to chronicity.

## 1. Introduction

Chikungunya is endemic in about 114 African, Asian, and American countries and territories, with sporadic outbreaks in other locations and an estimated number of annual symptomatic cases ranging between 52,774 and 328,943 [1]. In the Americas, the disease emerged in 2013, and in 10 years, the region had the highest global burden, with more than 3.7 million suspected cases reported [1]. Since 2016, Brazil has been the epicenter of Chikungunya epidemics in the Americas, with the highest number of reports, accounting for more than 1.5 million cases from 2016 to 2023 [2].

Chikungunya is a systemic infection caused by the Chikungunya virus (CHIKV) (Alphavirus genus, Togaviridae family), which is transmitted by mosquitoes of the Aedes genus, especially the species *Aedes aegypti*. There are four genotypes of CHIKV, namely West African, East/Central/South African (ECSA), Asian, and Indian Ocean [3].

The most typical manifestation of Chikungunya is intense and debilitating joint pain, usually affecting different sites, impacting daily activities, and resulting in mental distress [4]. In the acute phase of the disease, the patient can present with pain in the back, muscles, and joints; headache; joint swelling; rash; and fatigue. The chronic phase occurs when joint manifestations persist for three or more months, having periods of remission and recurrence, with 42.5% of Chikungunya cases developing arthritis or arthralgia, 25% inflammatory rheumatism, and some reporting musculoskeletal stiffness, with frequencies varying in different populations [1,4,5,6,7,8]. The frequencies of chronic symptoms may differ according to CHIKV lineages, with higher prevalence reported in the ECSA genotype [9].

The pathogenesis of chronic Chikungunya has not yet been fully clarified. Host characteristics could contribute to chronicity, such as female sex, aging, diabetes, hypertension, and severe pain in the acute stage [10]. The absence of specific drugs for treatment and licensed vaccines poses additional challenges for the disease’s clinical management and prevention [11].

In Brazil, the high prevalence of chronic arthralgia in Chikungunya infections demands long-term multidisciplinary medical care, burdening health systems and leading to social impacts [12]. Therefore, this study aims to identify the potential factors associated with chronic Chikungunya to improve the identification and clinical management of cases prone to chronicity.

## 2. Materials and Methods

### 2.1. Study Design

This case–control study involves data on Chikungunya reports in residents of Vitória municipality, Espírito Santo state, Brazil, with clinical manifestations beginning between 1 March 2016 and 31 December 2020. The outcome assessed was the progression to the chronic phase of Chikungunya. A total of 183 chronic cases included in the study were matched with 183 controls who did not develop chronic symptoms. The controls were selected by simple random sampling.

### 2.2. Study Area and Population

Vitória is Espírito Santo’s capital on southeast Brazil’s coast. It has 97.123 km^2^ of insular and continental areas and a tropical humid climate. The population in Vitória in 2022 was 322,869 people, with a population density of 3324.33 inhabitants per km^2^ [13].

### 2.3. Data Source

Data on Chikungunya compulsory reports from 1 March 2016 to 31 March 2020 were collected from the Notifiable Diseases Information System (SINAN) and, from 1 April 2020 to 31 December 2020, from the eSUS Health Surveillance (eSUS-VS). Data extraction occurred on 17 September 2021. In addition, the medical records from Rede Bem-Estar were accessed to follow patients’ clinical outcomes.

### 2.4. Inclusion and Exclusion Criteria

The study included laboratory-confirmed cases of Chikungunya in viral isolation, RT-PCR or ELISA IgM, who resided in Vitória. Reports without data on the Chikungunya outcome and duplicates were excluded.

### 2.5. Variables

The independent variables comprised age, sex, Chikungunya clinical manifestations, pre-existing diseases, and hospitalization. The outcome was chronic Chikungunya, characterized by the persistence of clinical manifestations for over three months. Clinical manifestations in the acute phase included fever, intense polyarthralgia, back and head pain, rash, and fatigue [14].

### 2.6. Sample Size

Considering a 95% confidence interval, a test power of 80%, an exposure in 75% of the controls, a proportion of one control for each case, and a three-fold difference in exposure between the groups, the minimum sample size was 113 cases and 113 controls. The calculation was performed using Statcalc (EpiInfoTM software version 7.2.2.6, Centers for Disease Control and Prevention, Atlanta, GA, USA).

### 2.7. Statistical Analysis

Categorical variables were described by absolute and relative frequencies and compared between the groups using Pearson’s chi-square test or Fisher’s exact test. Continuous variables were described as means and interquartile ranges and compared between the groups using the Mann–Whitney test. The variables with statistically significant differences were analyzed by binary logistic regression using the Forward Stepwise method. The results were presented as Odds Ratios (ORs) with a 95% confidence interval (95% CI), and a *p*-value ≤ 0.05 was considered significant. The analyses were performed using SPSS version 20.

### 2.8. Ethical Considerations

The Research Ethics Committee of the Federal University of Espírito Santo approved the study protocol under opinion number 4,393,656.

## 3. Results

Between March 2016 and December 2020, 3216 cases of Chikungunya with laboratory confirmation were identified in Vitória. Of these, 19 were excluded from the study—15 due to a lack of outcome data and 4 because they were duplicates. Thus, 3197 patients were eligible, of which, 3176 were confirmed by IgM serology in the first sample, 15 by IgM serology in the second sample, 15 by RT-PCR, and 1 by viral isolation. Some patients underwent more than one laboratory confirmation test. The data completeness of the variables in the included cases varied from 99.7% to 100%.

Of the eligible cases, 183 (5.7%) were classified as chronic; therefore, 183 non-chronic patients were randomly selected from the 3,014 who presented this classification. Most of the study population was female (73.2%), with ages ranging from 0 to 89 years and a median of 49.5 years (interquartile range = 37–61). The most frequent pre-existing diseases were arterial hypertension (31.1%) and diabetes (15.6%). Fever (89.6%), myalgia (89.6%), arthralgia (89.3%), and headache (82.0%) were the main Chikungunya clinical manifestations, and 2.6% of patients required hospitalization (Table 1).

Patients with chronic Chikungunya were significantly older than those who had non-chronic Chikungunya (*p*-value = 0.001); reported more hypertension (*p*-value = 0.042); and presented more nausea (*p*-value = 0.001), vomiting (*p*-value = 0.014), leukopenia (*p*-value = 0.014), and back pain (*p*-value = 0.057). Hospitalization (*p*-value = 0.095) was not associated with chronicity (Table 1).

In the multivariate analysis, nausea and leukopenia were associated with chronic presentation. On the other hand, there was a small protective effect for chronicity for age, which was lower among non-chronic patients (Table 2).

Binary logistic regression was carried out using the Forward Stepwise method. Variables not included in the model were vomiting, arterial hypertension, and back pain. X^2^ (3) = 28.588, *p*-value = 0.000, R^2^ Nagelkerke = 0.100, * Odds Ratio adjusted with 95% confidence interval. Sensitivity = 60.1%, specificity = 61.2%, accuracy = 60.7%, precision = 60.8%. F1-score = 60.4%, AUC = 0.6865.

## 4. Discussion

This study described the profile of those affected by Chikungunya, particularly regarding the evolution to chronicity and its associated factors. The demographic characteristics of those affected were like those in other settings in Brazil, with more reports of the disease in adults and women, representing the medical care-seeking behavior, not only the incidence [15]. Nevertheless, the influence of sex on immune response could have contributed to a higher proportion of Chikungunya-symptomatic females, with hormones affecting the production of proinflammatory cytokines and antibodies [16]. Aging was associated with chronicity, as previously reported, especially in patients over 40 years old [6,16]. Thus, age is a relevant factor for disease progression.

Arterial hypertension and diabetes were the most common pre-existing chronic diseases in Chikungunya cases since they are also the most frequent noncommunicable diseases in the Brazilian population. Both comorbidities were previously reported to affect 20% to 30% of patients with CHIKV infection [17]. In addition, hypertension was significantly more frequent in chronic Chikungunya, although not considerably significantly associated in the final model. Age may also confound this finding since hypertension is related to aging [18].

The main acute clinical manifestations of Chikungunya were fever, myalgia, arthralgia, and headache, comparable in frequency to those found in a systematic review that showed 92% of cases presenting swelling and joint pain, 85% fever, 52% myalgia, and 30% to 40% neurological symptoms, such as headache [19]. The CHIKV tropism to joints, muscles, bones, and nerves, accompanied by the release of inflammatory and pyrogenic cytokines, such as IL-6, IL1b, and TNFα, likely contributed to the emergence of these signs and symptoms [20]. Notably, we detected that chronic patients presented significantly more symptoms, including vomiting and back pain, and especially nausea and leukopenia, which were the most significantly associated factors for chronicity in the final model. These findings are consistent with previous studies demonstrating that severe pain in the acute phase of the disease predicts its chronicity [6,16]. Moreover, vomiting was associated with an increased risk of severity and death by CHIKV infection [21]. Leukopenia was also previously identified as a risk factor for hospitalization, severity, and deaths in patients with Chikungunya, although no previous studies linked it with chronic cases [21,22]. While it remains unknown how increased nausea and leukopenia during the acute phase are linked to the increased occurrence of chronic symptoms, one possible explanation may be related to the increased use of opioids to manage exacerbated pain that increases the side effects and promotes CHIKV disease pathogenesis.

Hospitalization was infrequent and not associated with chronic Chikungunya. In Vitória, hospitalization was previously related to specific age groups, such as those lower than two and over 65 years, and in patients with complications such as thrombocytopenia, diarrhea, and syncope [23]. Concerning the epidemiology of CHIKV in our municipality, the ECSA genotype was identified in 2016 and 2017. In other areas of Brazil, the Asian genotype was also identified [3,24]. Although we did not evaluate the viral genotypes that were circulating in Vitória in this study, the prevalence of chronic cases in ECSA is expected to vary from 40% to 60%, indicating a possibility of under-reporting of the condition in the municipality population and the necessity of improved follow-up of the patients and updating the report forms [9].

The study limitations are mainly related to secondary data from the compulsory reports and the possibility of information bias. Nevertheless, medical records were consulted to improve data quality and confirm the outcome. However, the health services did not conduct follow-up, and only patients who spontaneously returned to healthcare were reported as chronic. Therefore, we cannot discard the misclassification of patients considered non-chronic. The strict inclusion of laboratory-confirmed cases prevented the inclusion of other conditions with similar clinical manifestations, such as dengue [25,26]. In addition, we did not access other possible determinants of chronicity, such as viral and immunological factors. Cohort studies are suggested to confirm the presented findings, considering the follow-up of leukocytes and therapeutic approaches, such as the use of opioids. In addition, future prospective studies should also evaluate viral and immunological factors in Chikungunya chronicity, including how sex contributes to clinical manifestations.

This study reinforces the necessity of monitoring Chikungunya patients, especially those older, in need of opioid use due to severe pain or with laboratory alterations, such as leukopenia, for the better identification and clinical management of cases prone to chronicity.

## 5. Conclusions

Chronic Chikungunya was associated with older age, the presence of nausea, and leukopenia, suggesting the need for the longer-term monitoring of patients with these characteristics to improve diagnosis and treatment.

## Figures and Tables

**Table 1:** Characteristics of Chikungunya cases according to chronicity outcome.

Characteristic	Chronic n = 183 n (%)	Non-Chronic n = 183 n (%)	Total n = 366 n (%)	*p*-Value
**Sex**				
Female	140 (76.5%)	128 (69.9%)	268 (73.2%)	0.157 ^ƪ^
Male	43 (23.5%)	55 (30.1%)	98 (26.8%)	
**Age**				
Median (interquartile range)	53 (41–61)	46 (31–61)	49.5 (37–61)	0.006 ^#^
**Pre-existing diseases**				
Diabetes	33/182 (18.1%)	24 (13.1%)	57 (15.6%)	0.187 ^ƪ^
Liver disease	5/182 (2.7%)	1 (0.5%)	6 (1.6%)	0.121 ^¶^
Arterial hypertension	66 (36.1%)	48 (26.2%)	114 (31.1%)	0.042 ^ƪ^
Acid-peptic disease	6 (3.3%)	3 (1.6%)	9 (2.5%)	0.502 ^¶^
**Clinical manifestation**				
Fever	163 (89.1%)	165 (90.2%)	328 (89.6%)	0.732 ^ƪ^
Myalgia	162 (88.5%)	166 (90.7%)	328 (89.6%)	0.493 ^ƪ^
Headache	154 (84.2%)	146 (79.8%)	300 (82.0%)	0.277 ^ƪ^
Rash	66 (36.1%)	68 (37.2%)	134 (36.6%)	0.828 ^ƪ^
Vomiting	42 (23.0%)	24 (13.1%)	66 (18.0%)	0.014 ^ƪ^
Nausea	107 (58.5%)	74 (40.4%)	181 (49.5%)	0.001 ^ƪ^
Back pain	133 (72.7%)	116 (63.4%)	249 (68.0%)	0.057 ^ƪ^
Conjunctivitis	19 (10.4%)	22 (12.0%)	41 (11.2%)	0.619 ^ƪ^
Arthritis	90 (49.2%)	86 (47.0%)	176 (48.1%)	0.676 ^ƪ^
Arthralgia	166 (90.7%)	161 (88.0%)	327 (89.3%)	0.397 ^ƪ^
Petechiae	26 (14.2%)	30 (16.4%)	56 (15.3%)	0.561 ^ƪ^
Leukopenia	37 (20.2%)	20 (10.9%)	57 (15.6%)	0.014 ^ƪ^
Positive tourniquet test	26 (14.2%)	28 (15.3%)	54 (14.8%)	0.768 ^ƪ^
Retroorbital pain	82 (44.8%)	72 (39.3%)	154 (42.1%)	0.290 ^ƪ^
**Hospitalization**	7 (4.2%)	2 (1.1%)	9 (2.6%)	0.095 ^¶^

^ƪ^ Pearson’s Chi-square test, ^¶^ Fisher’s exact test, ^#^ Mann–Whitney test.

**Table 2:** Characteristics associated with the chronic Chikungunya.

Characteristic	OR *	95% Confidence Interval	
		Lower Limit	Upper Limit
**Age**	0.979	0.968	0.991
**Nausea**	2.109	1.374	3.238
**Leukopenia**	2.060	1.122	3.779

## Data Availability

The data presented in this study are available on request from the corresponding author due to the presence of patient data.
